# Definition of compartment-based radical surgery in uterine cancer: modified radical hysterectomy in intermediate/high-risk endometrial cancer using peritoneal mesometrial resection (PMMR) by M Höckel translated to robotic surgery

**DOI:** 10.1186/1477-7819-11-198

**Published:** 2013-08-16

**Authors:** Rainer Kimmig, Bahriye Aktas, Paul Buderath, Pauline Wimberger, Antonella Iannaccone, Martin Heubner

**Affiliations:** 1 Department of Gynecology and Obstetrics, West German Cancer Center, University Clinic Essen, University of Duisburg-Essen, Hufelandstrasse 55, Essen, 45147, Germany; 2 Department of Gynecology and Obstetrics, University Hospital Carl Gustav Carus, TU Dresden, Fetscherstrasse 74, Dresden, 01307, Germany

## Abstract

**Background:**

The technique of compartment-based radical hysterectomy was originally described by M Höckel as total mesometrial resection (TMMR) for standard treatment of stage I and II cervical cancer. However, with regard to the ontogenetically-defined compartments of tumor development (Müllerian) and lymph drainage (Müllerian and mesonephric), compartments at risk may also be defined consistently in endometrial cancer. This is the first report in the literature on the compartment-based surgical approach to endometrial cancer. Peritoneal mesometrial resection (PMMR) with therapeutic lymphadenectomy (tLNE) as an ontogenetic, compartment-based oncologic surgery could be beneficial for patients in terms of surgical radicalness as well as complication rates; it can be standardized for compartment-confined tumors. Supported by M Höckel, PMMR was translated to robotic surgery (rPMMR) and described step-by-step in comparison to robotic TMMR (rTMMR).

**Methods:**

Patients (n = 42) were treated by rPMMR (n = 39) or extrafascial simple hysterectomy (n = 3) with/without bilateral pelvic and/or periaortic robotic therapeutic lymphadenectomy (rtLNE) for stage I to III endometrial cancer, according to International Federation of Gynecology and Obstetrics (FIGO) classification. Tumors were classified as intermediate/high-risk in 22 out of 40 patients (55%) and low-risk in 18 out of 40 patients (45%), and two patients showed other uterine malignancies. In 11 patients, no adjuvant external radiotherapy was performed, but chemotherapy was applied.

**Results:**

No transition to open surgery was necessary. There were no intraoperative complications. The postoperative complication rate was 12% with venous thromboses, (n = 2), infected pelvic lymph cyst (n = 1), transient aphasia (n = 1) and transient dysfunction of micturition (n = 1). The mean difference in perioperative hemoglobin concentrations was 2.4 g/dL (± 1.2 g/dL) and one patient (2.4%) required transfusion. During follow-up (median 17 months), one patient experienced distant recurrence and one patient distant/regional recurrence of endometrial cancer (4.8%), but none developed isolated locoregional recurrence. There were two deaths from endometrial cancer during the observation period (4.8%).

**Conclusions:**

We conclude that rPMMR and rtLNE are feasible and safe with regard to perioperative morbidity, thus, it seems promising for the treatment of intermediate/high-risk endometrial cancer in terms of surgical radicalness and complication rates. This could be particularly beneficial for morbidly obese and seriously ill patients.

## Background

Treatment of endometrial cancer is primarily surgical. However, there is ongoing debate on the extent of surgical radicalness, predominantly with regard to lymphadenectomy (LNE). In addition, adjuvant radiotherapy has been shown to improve local control but does not increase survival probability. Indeed, adjuvant chemotherapy seems to increase survival probability in patients with high-risk endometrial cancer [1,2]. Surgical treatment of endometrial cancer can be performed with lower risk but apparently unaltered oncological efficiency by minimally invasive surgery. This also holds true for minimally invasive approaches and robotic surgery [3-6].

Although there are many reports of the results of different surgical approaches, a standardization of surgical treatment for intermediate/high-risk endometrial cancer is missing. Standardization is crucial for the evaluation of locoregional tumor control by surgery alone, as it has been proposed for cervical cancer [7-9]. Applying the findings of basic research on boundary formation and maintenance in tissue development [10], as well as the results of clinical research on ontogenetic compartments of cancer spread in endometrial cancer [11], the compartments of local and regional cancer spread can be defined and surgical techniques adapted to remove the compartments at risk entirely. In analogy to cervical cancer, this may also impact upon locoregional cancer recurrence in endometrial cancer without any additional adjuvant radiotherapy. As proposed by M Höckel, the technique of TMMR can be modified and adapted to localization and lymph drainage of endometrial cancer, with regard to the paramesonephric-mesonephric-Müllerian tubercle complex-derived tissue compartments (personal communication).

In this paper, we describe the resulting technique of peritoneal mesometrial resection (PMMR), which has been translated from open to robotic surgery (rPMMR). Clinical outcome data are reported first.

## Methods

### Surgical technique

The training of the first author, the development and description of the surgical technique of robotic total mesometrial resection (rTMMR) and periaortic robotic therapeutic lymphadenectomy (rtLNE) have recently been described [12,13]. In close cooperation with M Höckel, the method of rPMMR has been developed and surgical steps to remove ontogenetically-derived compartments of permissive tumor progression have been defined. Although endometrial cancer also arises within the Müllerian compartment, there are two important differences to cervical cancer with regard to tumor site and compartment-related tumor progression. It is important to note that the technique of PMMR and therapeutic lymphadenectomy (note: “therapeutic” means “therapeutically intended”) (tLNE) is deduced from the research on embryonic development of organ compartments and stringent control of compartment borders; however, it has not yet been proven by clinical studies.

**Figure 1:**
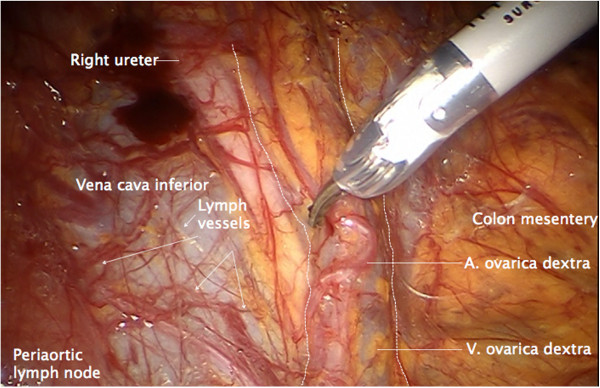
**Right infundibulopelvic ligament.** Arteria ovarica and vena ovarica, the border to the right colon mesentery, and the lymphatic anastomosis to the right inframesenteric periaortic lymph nodes (arrows) are visible.

The first difference is that there is an additional downstream lymphatic drainage along the mesonephric-derived ovarian vessel system into the periaortic nodes (Figure 1).

The second difference is that there is no drainage into the deep preischiadic nodes via the ligamentous (fibrofatty) mesometria, as long as there is no distal cervical stromal infiltration.

On the other hand, the drainage along the vascular mesometrium is almost identical to cervical cancer, using the same lymph channels along the uterine vessels into the external, internal and common iliac nodes.

Thus, with regard to the technique of radical hysterectomy, the vascular mesometrium should be resected in endometrial cancer almost identically to cervical cancer. However, the ligamentous mesometrium, that is sacrouterine and rectovaginal ligaments, may be left *in situ* if there is no cervical involvement. Furthermore, it is important to remove the vascular anastomoses between the uterine and ovarian vessel system, to guarantee clearance of the whole Müllerian and mesonephric compartment at risk. This can be achieved by *en bloc* resection of the entire broad ligament together with the peritoneum covering this tissue region. With regard to locoregional spread, complete bilateral resection of adnexa and infundibulopelvic ligaments including intercalated lymph nodes is mandatory. With regard to parietal LNE, pelvic and periaortic nodes represent primary basins, thus implying a complete bilateral pelvic and periaortic LNE in case of therapeutic intent as previously described [12]. As a consequence of the lack of lymph drainage via the ligamentous mesometria in endometrial cancer, the deep preischiadic nodes may be left *in situ*.

The resulting technique of rPMMR will be described in detail, step-by-step. Particular steps are identical to rTMMR and the accordant technique has been previously described [13].

### Patients

As a first proof of feasibility, 39 out of 42 patients with stage I to III endometrial cancer, according to International Federation of Gynecology and Obstetrics (FIGO) classification, were treated by rPMMR with or without rtLNE; 3 out of 42 patients with low-risk endometrial cancer underwent robotically-assisted, non-modified total laparoscopic hysterectomy (TLH). The median age of patients was 59 years (range 26 to 81 years) and the mean body mass index (BMI) was 31 kg/m^2^ (range 18 to 57 kg/m^2^). There were 40 patients with endometrial carcinoma (endometroid, 34 patients; serous, three patients; clear cell, one patient; adenosquamous, one patient; and squamous, one patient), one patient with a mixed Müllerian tumor and one patient with a rhabdomyosarcoma. FIGO stages of endometrial carcinomas were distributed: stage IA, 21 patients; stage IB, ten patients; stage II, three patients; stage IIIA, one patient; and stage IIIC, five patients. There were five patients with grade I, 25 patients with grade II and ten patients with grade III tumors. Four tumors showed lymphangiosis.

tLNE was performed by robotic surgery in analogy to the procedure described by Höckel [9,11] and Kimmig *et al.*[12]. None of the patients with grade I tumors underwent LNE. Six patients presented with nodal metastasis, and the number of positive nodes was 1, 1, 2, 3, 5 and 24, respectively. The total number of nodes removed is shown in Results. However, with regard to compartmental-defined radical surgery, complete clearance of lymph basins and connecting intercalated nodes downstream to the tumor was defined as a superior quality parameter to the number of nodes. Intercalated nodes are embryonically ‘immigrated’ nodes connecting the lymph system of the organ compartment to the lymph basins. For endometrial cancer (uterine pathway), these include: intercalated mesometrial nodes (mm), paravisceral nodes (internal iliac nodes excluding gluteal and rectal nodes) (pv), external iliac nodes (ei), common iliac nodes (ci) and presacral/subaortic nodes (ps) on both sides. Additionally, surgery was extended following the lymphatic ovarian drainage including the ovarian pathway and intercalated infundibulopelvic nodes (ifp), and periaortic nodes including inframesenteric nodes (im) and supramesenteric/infrarenal nodes (sm/ir). Perioperative morbidity and early postoperative morbidity were analyzed. In addition, frequency of perioperative blood transfusions and differences in perioperative hemoglobin concentrations were noted. The tumor-related outcome was recorded.

### Statistical analysis

Analysis of clinical and histopathological data was performed using SPSS (Chicago, IL, USA) version 17.0 for Macintosh. Considering the limited number of patients and the explorative character of this study, we conducted a descriptive analysis only.

### Technique and results

#### Preparation of robotically-assisted laparoscopy and trocar positioning

Patients were placed in the Trendelenburg position with at least 25 to 30 degrees, and with side-docking of the patient cart. The trocars were positioned with the camera trocar approximately 5 to 10 cm supra-umbilically and two lateral robotic trocars approximately 5 to 10 cm above the upper anterior iliac spine on both sides, that is, one additional robotic trocar on the left between the camera trocar and the left lateral trocar, and one assistant trocar of 10 mm diameter on the right side between camera trocar and right lateral trocar. At least 10 cm between the trocar incisions assured free and adequate mobility. Before rPMMR, bilateral pelvic and periaortic rtLNE were performed as outlined in Kimmig *et al.*[12].

Concomitantly to the removal of lymph nodes, the infundibulopelvic ligaments containing lymph vessels and intercalated nodes were completely exposed and totally resected. The anastomoses to the periaortic lymphatic system, at the level of the aorta, caval vein and renal veins, were kept intact and mobilized without separation. All regional nodes were then removed, except for the intercalated mesometrial nodes located predominantly in the vascular mesometrium.

**Figure 2:**
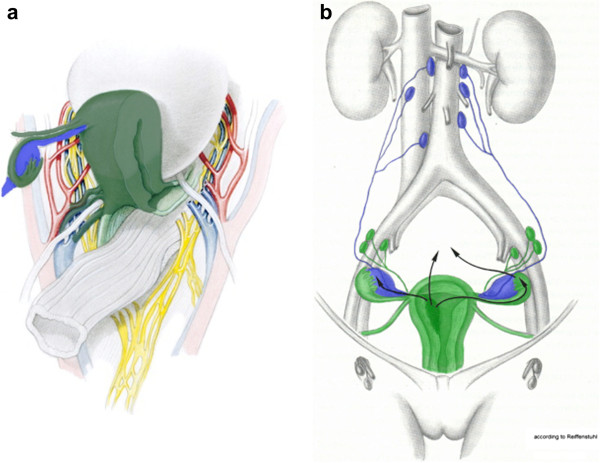
**The structures of the female genital tract with reference to the embryologic Müllerian and mesonephric compartment.** The accordant structures resected in endometrial cancer are colored dark green for the Müllerian Compartment **(a)** and blue for the mesonephric lymphatic drainage **(b)**, ovarian and infundibulopelvic system). The parts of the Müllerian compartment that are only resected in stage II or III disease are colored light green. Modified from Höckel [14] and Hepp *et al.*[15], with permission of Elsevier and Urban & Schwarzenberg, respectively.

Prior to the standardized description of rPMMR, the principles and nomenclature of the technique by M Höckel should be kept in mind. As shown in Figure 2a, the Müllerian compartment consists of the uterus, fallopian tubes and vascular mesometria/mesocolpia; corresponding to the tissue surrounding and accompanying the uterine vessels to the iliac vessels, and the vesicouterine ligaments anteriorly and ligamentous mesometria dorsally; corresponding to the fibrofatty sacrouterine and rectouterine/vaginal ligaments, and the proximal vagina. According to the technique, all of these structures should be completely removed, except for the ligamentous mesometria and the vagina with wide tumor-free margins. Nevertheless, the inferior hypogastric plexus should be identified in order to safely separate it from the vascular mesometrium. Additionally, the intercalated tissue between the Müllerian-derived uterine vessel system and the ovarian vessel system of mesonephric origin should be removed entirely, together with the ovary and the ovarian vessel system due to the lymphatic drainage of the uterine corpus (Figure 2b).

The procedure of rPMMR and rtLNE starts by opening the peritoneum laterally to the right infundibulopelvic ligament at the level of the right common iliac artery.

#### Step 1

First, the peritoneum is incised laterally to the right iliac common artery and the right infundibulopelvic ligament, and divided cranially to expose the anterior aspect of the vena cava, the right ureter, the right infundibulopelvic ligament and the right colon mesentery. Usually, lymphatic anastomoses between the infundibulopelvic ligament and the low right periaortic nodes can be identified (Figure 1).

#### Step 2

**Figure 3:**
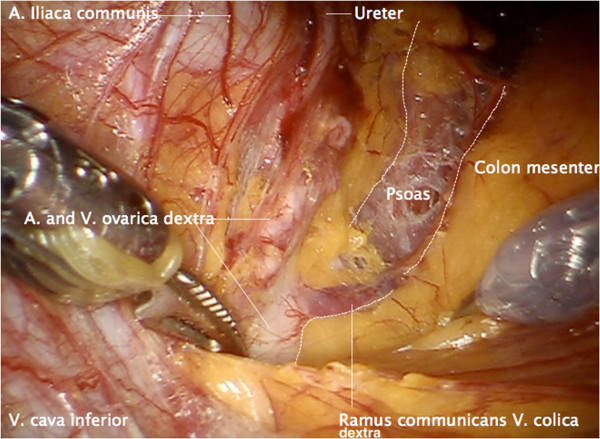
Separation of the right infundibulopelvic ligament from the right mesocolon and dissection of connecting vessels.

The border between the mesonephric compartment of the infundibulopelvic ligament and the right colon mesentery is identified, and the structures are separated. Anastomosing vessels are identified and sealed (Figure 3).

#### Step 3

**Figure 4:**
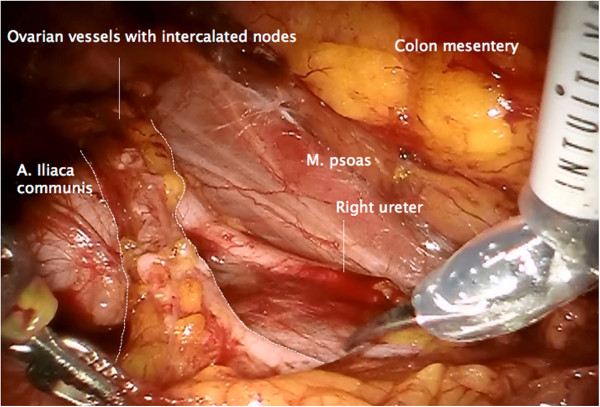
**Mobilization of the right infundibulopelvic ligament from laterally to medially and resection together with adherent periaortic nodes ventrally of the vena cava.** The ureter and the vessels supplying the mesureter are preserved and mobilized.

The infundibulopelvic ligament is elevated and dissected from laterally to medially, keeping the connections to the periaortic lymphatic system intact, and separating it from the right ureter and mesureter dorsally (Figure 4).

#### Step 4

**Figure 5:**
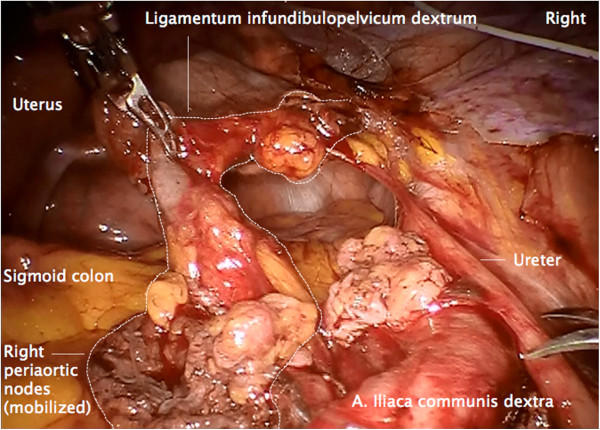
**Dissection of the ovarian artery and ovarian vein, at the aortic and cava level, respectively.** The right periaortic nodes are left attached and represent the first draining nodes (compare to Figure 2b).

Resection of the right infundibulopelvic ligament, following the dissection of ovarian vessels at their origin together with periaortic LNE, is performed completely up to the renal vessels, including the nodes located between and dorsally of the vena cava and aorta as previously described [12]. The right infundibulopelvic lymphatic drainage is completely mobilized together with adherent right periaortic nodes (Figure 5).

#### Step 5

**Figure 6:**
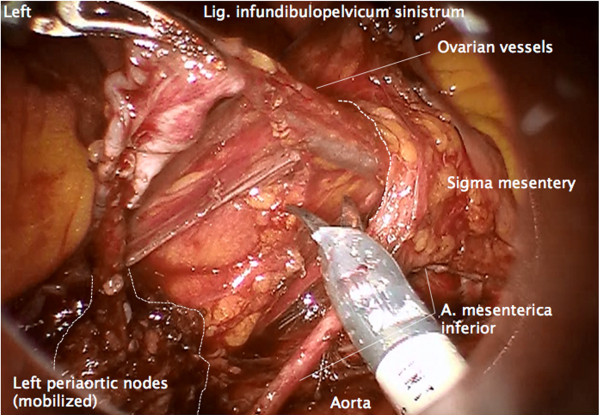
**Same preparation of the left side.** Following dissection of the ovarian vessels at their aortic and renal origin on the left, the infundibulopelvic ligament is dissected from the left mesocolon and followed through a prepared channel dorsally to the mesosigmoid. The left infrarenal periaortic nodes are kept in continuity to the ovarian vessel system as shown on the right.

**Figure 7:**
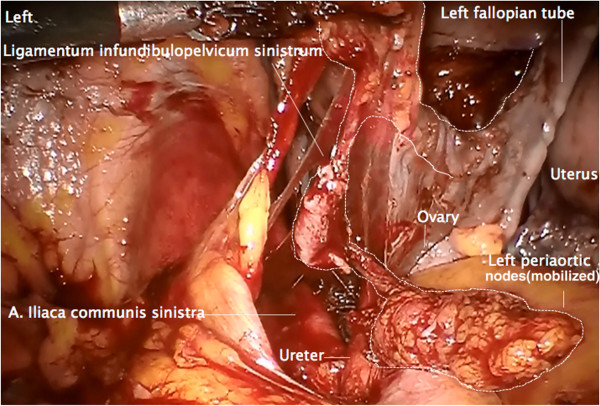
**Transferral of the left infundibulopelvic ligament.** Following complete mobilization, the left infundibulopelvic ligament is pulled through the sigmoid ‘tunnel’ to the pelvis together with the left infrarenal periaortic nodes, to guarantee complete resection of the mesonephric ovarian drainage and avoiding division of the ligament.

Together with the left periaortic node dissection, the left ovarian vein and artery are resected at their origin [12], and mobilized laterally and dorsally to the left colon mesentery and the left ureter, identically to the right (Figure 6). Along the vessels, a channel dorsally to the sigmoid mesentery is prepared to the left pelvis at the level of the left common iliac artery. The infundibulopelvic ligament together with the adherent infrarenal periaortic nodes is then transferred to the pelvis through this channel (Figure 7).

#### Step 6

Completion of the pelvic rtLNE as previously described for endometrial cancer [12].

#### Step 7

**Figure 8:**
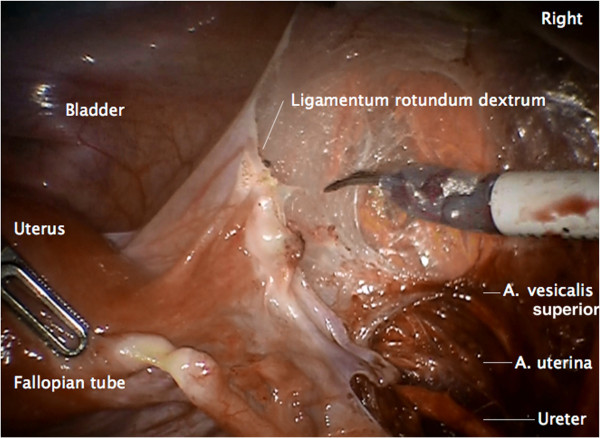
**Preparation of vascular anastomoses of the uterine and ovarian vessel system containing the accordant lymph drainage.** Lateral peritoneal incision with preparation of the lateral aspect of the vascular mesometrium and division of the round ligament.

**Figure 9:**
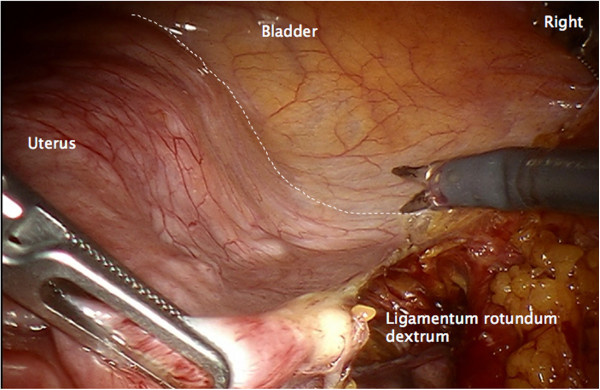
Ventral incision line to preserve the peritoneal covering the uterine and ovarian vessel system ensures complete resection.

**Figure 10:**
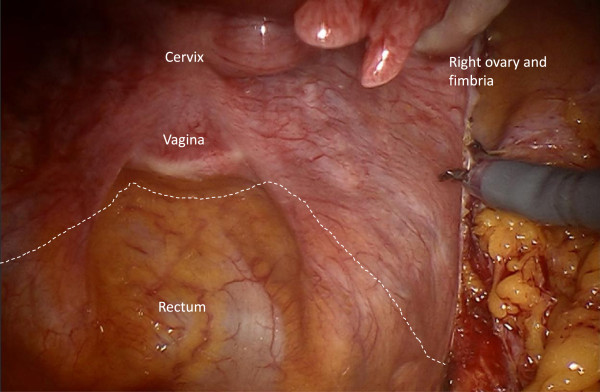
Dorsal incision line caudally of the uterine vessels identified through the intact peritoneum.

Incision of the peritoneum covering the round ligament, the ovarian vessels, the utero-ovarian junctions and the vascular mesometrium is performed. This maneuver assures complete resection of both regions together with the ovaries and the connecting structures (Figures 8, 9, 10).

#### Step 8

Radical hysterectomy with complete resection of the vascular mesometrium is performed as described in detail for rTMMR [13]. However, the separation from its conjunction with the adnexa and infundibulopelvic ligaments can be omitted. In endometrial cancer without cervical infiltration, the resection of ligamentous mesometria can also be omitted.

#### Step 9

The same procedure is undertaken on the left side. As in endometrial cancer, the tumor primarily arises in the Müllerian compartment and it has to be demonstrated that the vaginal wall (belonging to the same ontogenetic compartment) at the level of resection presents definite clear margins. Closure of the vagina can be undertaken by running suture.

**Figure 11:**
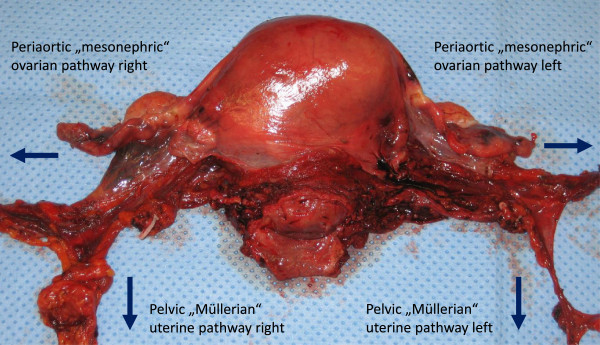
**Specimen of the uterus (ventral aspect) following rPMMR and rtLNE, showing the completely resected uterine and ovarian vessel system, without separation of connecting vessels and surrounding tissue.** The uterine and ovarian pathways of lymph drainage are indicated by arrows. rPMMR, robotic peritoneal mesometrial resection; rtLNE, therapeutic lymphadenectomy.

**Figure 12:**
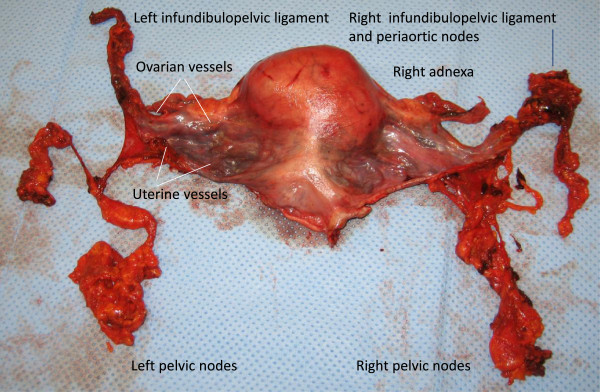
Same specimen of the uterus (dorsal aspect) together with the pelvic nodes, kept in continuity with the uterine drainage system.

A postoperative specimen following rPMMR and rtLNE in high-risk stage I endometrial cancer is shown in Figures 11 and 12. The complex of vascular mesometrium connected to the mesonephric infundibulopelvic vessel system, responsible for the different pelvic and periaortic pathways to the primary lymph basins, can clearly be identified (Figure 11). *En bloc* resection with the overlaying peritoneum facilitates the complete and intact removal of these connections (Figures 11 and 12). Connections to the pelvic and periaortic lymph basins can also be preserved when intended.

## Results

In total, 42 patients received robotically-assisted laparoscopic surgery. Thirty-nine patients received rPMMR with bilateral salpingo-oophorectomy (n = 12), additional pelvic tLNE (n = 8) or pelvic and periaortic LNE (n = 19). Three patients underwent completion of surgery following hysterectomy and diagnosis of endometrial cancer including resection of mesometria. Infundibulopelvic ligaments and tLNE were categorized as rPMMR with tLNE. Another two patients received immediate secondary robotic surgery for periaortic LNE in case of clinically inconspicuous but histologically verified positive pelvic nodes, and were also categorized as rPMMR with pelvic and periaortic tLNE. In six cases, resection of ligamentous mesometria was added due to suspicion of additional cervical involvement. The remaining three patients received robotic total laparoscopic hysterectomy (rTLH) only for stage IA endometrial cancer.

All operations were completed endoscopically without switching to open surgery. No intraoperative complications occurred. All tumors were histologically resected R0, but one tumor was diagnosed Rx (pT3b, pN1 (24/46)). Mean lymph node count was 33 (± 11) and 35 (± 10) for pelvic and pelvic/periaortic LNE, respectively. Overall, the mean decrease (± SD) of perioperative hemoglobin concentration (on the first postoperative day) was 2.4 g/dL (± 1.3 g/dL), with the lowest difference in rTLH (0.7 g/dL (± 0.6 g/dL)). The decrease following rPMMR was 2.1 g/dL (± 1.0 g/dL), with pelvic tLNE 2.6 g/dL ± 1.1 g/dL; essentially the same for pelvic and periaortic tLNE accounting for 2.6 g/dL ± 1.5 g/dL. Only one patient required transfusion following rPMMR and pelvic tLNE, with a preoperative hemoglobin concentration of 9.0 g/dL (2.4%).

Postoperative complications were relatively rare and corresponded to a total complication rate of 12%. One patient had superficial vein thrombosis, another patient had deep vein thrombosis, one patient developed an infected lymph cyst, another patient developed transient postoperative aphasia (all following rPMMR with pelvic and periaortic tLNE), and one patient had transient dysfunction of micturition following rPMMR only.

Endometrial cancer patients with stage III tumors, serous histology and/or positive lymph nodes were treated with six cycles of adjuvant platinum-based chemotherapy (n = 11). No patients received adjuvant external beam radiotherapy.

Two patients with endometrial cancer developed recurrent disease. The first patient with endometrial cancer recurrence was a 26-year-old woman with obesity (BMI 57 kg/m^2^). This was the only patient who required transfusion, showing a preoperative hemoglobin concentration of 9.0 g/dL. The initial tumor stage was pT1a, N0 (0/29), G3 and the histopathology revealed an endometrioid tumor type. The patient did not receive adjuvant treatment. One year after initial diagnosis, the patient presented with liver metastases and there was no evidence of relapse in the pelvis. The second patient with endometrial cancer recurrence initially had a tumor stage of pT3b, N1 (24/46), G2 (endometrioid histopathology). The tumor was completely resected macroscopically, but was shown to be histologically Rx, indicating that the tumor had already transgressed compartment borders. This patient received adjuvant chemotherapy with carboplatin (area under curve (AUC) 5) and paclitaxel 175 mg/m^2^ d1, q3w over six cycles. After 11 months, the patient presented with local pelvic recurrence and liver metastases, and was treated with palliative chemotherapy. Both patients died from metastatic disease during the observation period. There was no further recurrence or deaths during follow-up. At present, recurrence-free survival is 95% (40 out of 42 patients).

## Discussion

Currently, there is ongoing international debate about the extent of surgery, mode of access and correct choice of adjuvant treatment for intermediate/high-risk endometrial cancer [1-6]. Despite prospective data showing no benefit of adjuvant irradiation therapy, with regard to overall survival for patients with endometrial cancer, no relevant change in clinical routine has been observed, at least following the first publications of the Gynecologic Oncology Group-Adjuvant Radiation for Intermediate Risk Endometrial Cancers (GOG99) and Post Operative Radiation Therapy in Endometrial Carcinoma (PPORTEC1) [16]. Thus, highly effective and potentially curative radiotherapy will be wasted in the adjuvant setting, and will not be available for treatment in cases of recurrent disease.

With regard to the recurrence pattern of endometrial cancer, approximately 30 to 50% of patients experience locoregional recurrence, while the remaining patients present with distant or concomitant distant and locoregional recurrence [17-19]. Locoregional recurrences usually arise in the vagina as central pelvic recurrence in the Müllerian compartment or at the pelvic side wall, typically in pelvic lymph nodes. Prognosis is usually poor except for the isolated vaginal recurrence [20]. There is some evidence that more radical surgery may lower locoregional recurrence, not only in the pelvis [17,21] but also with respect to periaortic nodes [22,23]. However, it is difficult to compare results from clinical studies due to the large variety of surgical strategies and quality.

Thus, a standardization and clear definition of surgical procedures is urgently required, since surgery is without doubt the mainstay in the treatment of primary endometrial cancer. Data regarding LNE vary grossly between different studies. If pelvic and periaortic LNE is performed to provide regional cure by itself, then it should, at least intentionally, be complete. Additionally, the compartments at risk of lymph basins to be removed need to be exactly defined.

Regarding tLNE, we recently proposed a surgical strategy demonstrated by minimally invasive, robotically-assisted surgery [12]. On the basis of preclinical and clinical findings of ontogenetically-defined boundary formation and strict border control [10], and the clinical research on ontogenetic compartments of cancer spread in gynecological cancer [11], the local and regional compartments at risk in endometrial cancer can be defined as they have been for cervical cancer. As outlined already, there is no need for resection of the lymphatic drainage region of the lower cervical Müllerian compartment in endometrial cancer if the tumor is confined to the uterine corpus. Additionally, lymph drainage of the uterine corpus follows the ovarian vessels of the mesonephric origin directly to the periaortic nodes. Consequently, these compartments need to be cleared by tLNE in endometrial cancer.

If M Höckel’s concept of locoregional control by compartment-based surgery alone holds true for endometrial cancer, then the implementation of ontogenetic anatomy in surgical strategies is crucial to achieve best results in terms of local and regional tumor control. Following translation to robotic surgery, we demonstrate in a first feasibility analysis that this method may be reproduced and safely performed with regard to perioperative morbidity, irrespective of additional morbidity and BMI of patients. Interestingly, there was no remarkable difference in blood loss between the different approaches of rPMMR with or without LNE. However, postoperative complications were more frequent in the group with periaortic tLNE, which not surprisingly indicates that extension of surgery and operation time play a role in postoperative morbidity. There was not a single isolated locoregional recurrence despite omitting external beam radiotherapy generally.

Since the follow-up time of the patients is limited, at present we cannot conclude that PMMR and rtLNE exert excellent locoregional control without additional radiotherapy; however, up until now, the postoperative course does not seem to disprove it. Furthermore, rPMMR and rtLNE were accompanied by relatively low morbidity, with regard to the patients’ preoperative condition, BMI and surgical radicalness. Due to the excellent three-dimensional high-definition (3DHD) vision, and the high precision and control of movements, this technique can be considered outstandingly suited for complex surgery maintaining a minimally invasive approach. It is evident that at least equal radicalness may be achieved compared to open surgery. The possibility of video documentation may be beneficial concerning scientific, educational and forensic aspects.

From our experience of this first series of rPMMR with and without rtLNE in endometrial cancer, compartment-based surgery appears in an oncological manner to exert excellent locoregional control combined with a low complication rate, especially with regard to preexistent morbidity of patients.

## Conclusions

In conclusion, we suggest that the minimally invasive approach of compartment-based oncologic surgery for endometrial cancer by robotic assistance is feasible and safe, with regard to perioperative morbidity for patients with locally-confined endometrial cancer. Herewith, we present a proposal for compartment-based, robotically-assisted surgery for intermediate/high-risk endometrial cancer with the intention of locoregional control by surgery alone. The next step will be to evaluate whether we can achieve comparable results in endometrial cancer patients to the excellent monocentric data of M Höckel in cervical cancer patients [7-9]. Certainly, a greater number of patients, a longer follow-up time and a multicenter approach will be mandatory for sufficient analysis. This standardized and reproducible surgical protocol could be the basis for evaluation with regard to locoregional control by surgery alone, without additional radiotherapy. Reflecting that the majority of patients who will die from endometrial cancer are also diagnosed with lung and liver metastases [24], this collective of comprehensively and reproducibly staged patients may be appropriate for the most important further development of systemic treatment for distant control not compromised by concomitant radiotherapy [2]. Finally, other surgical gynecologic oncologists may refer to this technique to standardize and compare surgical results of compartment-related surgery in the treatment of intermediate/high-risk endometrial cancer.

## Consent

Written informed consent was obtained from the patient for the publication of the data and accompanying images.

## Abbreviations

AUC: Area under curve; BMI: Body mass index; ci: Common iliac nodes; ei: External iliac nodes; GOG99: Gynecologic Oncology Group-Adjuvant Radiation for Intermediate Risk Endometrial Cancers; im: Inframesenteric nodes; ifp: Infundibulopelvic nodes; FIGO: International Federation of Gynecology and Obstetrics; LNE: Lymphadenectomy; mm: Mesometrial nodes; pv: Paravisceral nodes; PMMR: Peritoneal mesometrial resection; PORTECI: Post Operative Radiation Therapy in Endometrial Carcinoma; ps: Presacral/subaortic nodes; rPMMR: Robotic peritoneal mesometrial resection; rtLNE: Robotic therapeutic lymphadenectomy; rTLH: Robotic total laparoscopic hysterectomy; rTMMR: Robotic total mesometrial resection; sm/ir: Supramesenteric/infrarenal nodes; tLNE: Therapeutic lymphadenectomy; 3DHD: Three-dimensional high-definition; TLH: Total laparoscopic hysterectomy; TMMR: Total mesometrial resection.

## Competing interests

The authors declare that they have no conflicts of interest. RK received expense allowances/honoraria from Intuitive Surgical, Inc (Sunnyvale, CA, USA) from 2011 to 2013 for educational work in robotic surgery and training of gynecologic departments in Western Europe (proctoring).

## Authors’ contributions

RK contributed to conception and design of the analysis, analysis and interpretation of data, and drafting and finalization of the manuscript. BA contributed to conception, acquisition and analysis of data, and critically revising and drafting the manuscript. PB contributed to acquisition of data and drafting the manuscript. PW contributed to acquisition of data and critically revising the manuscript. AI contributed to acquisition and statistical analysis of data, and drafting the manuscript. MH contributed to the conception and design of the analysis, data interpretation, and critically revising and drafting the manuscript. All authors read and approved the final manuscript.
